# Empirical Analysis and Modeling of Stop-Line Crossing Time and Speed at Signalized Intersections

**DOI:** 10.3390/ijerph14010009

**Published:** 2016-12-23

**Authors:** Keshuang Tang, Fen Wang, Jiarong Yao, Jian Sun

**Affiliations:** 1 Department of Transportation Information and Control Engineering, College of Transportation Engineering, Tongji University, Shanghai 201804, China; tang@tongji.edu.cn (K.T.); JoanYao@tongji.edu.cn (J.Y.); 2 School of Urban Rail Transportation, Shanghai University of Engineering Science, Shanghai 201620, China; wangf_2016@163.com; 3 Department of Traffic Engineering, College of Transportation Engineering, Tongji University, Shanghai 201804, China; 4 Jiangsu Province Collaborative Innovation Center of Modern Urban Traffic Technologies, SiPaiLou #2, Nanjing 210096, China

**Keywords:** high-speed intersections, safety, flashing green, stop-line crossing behavior, multinomial logit model, 081202

## Abstract

In China, a flashing green (FG) indication of 3 s followed by a yellow (Y) indication of 3 s is commonly applied to end the green phase at signalized intersections. Stop-line crossing behavior of drivers during such a phase transition period significantly influences safety performance of signalized intersections. The objective of this study is thus to empirically analyze and model drivers’ stop-line crossing time and speed in response to the specific phase transition period of FG and Y. High-resolution trajectories for 1465 vehicles were collected at three rural high-speed intersections with a speed limit of 80 km/h and two urban intersections with a speed limit of 50 km/h in Shanghai. With the vehicle trajectory data, statistical analyses were performed to look into the general characteristics of stop-line crossing time and speed at the two types of intersections. A multinomial logit model and a multiple linear regression model were then developed to predict the stop-line crossing patterns and speeds respectively. It was found that the percentage of stop-line crossings during the Y interval is remarkably higher and the stop-line crossing time is approximately 0.7 s longer at the urban intersections, as compared with the rural intersections. In addition, approaching speed and distance to the stop-line at the onset of FG as well as area type significantly affect the percentages of stop-line crossings during the FG and Y intervals. Vehicle type and stop-line crossing pattern were found to significantly influence the stop-line crossing speed, in addition to the above factors. The red-light-running seems to occur more frequently at the large intersections with a long cycle length.

## 1. Introduction

Despite the availability of traffic control devices and traffic signal warrants in China, the lack of a universal signal timing guideline results in inappropriate or improper signal timings arbitrarily matching with different signal control devices. Among such signal control implementations by local authorities, the most widely used one is a pattern of a flashing green (FG) of 3 s, followed by a constant yellow time (Y) of 3 s and an all-red time (AR) of 1 s or 2 s. This pattern serves as a transition period between different phases and the duration of AR mainly depends on the intersection size [1,2]. Regardless of speed limits, the application of a constant 3 s of Y is actually inadvisable for it is supported that a 3 s of Y may be too short for approaching drivers to avoid dilemma zone (DZ) at the intersections with speed limits higher than 50 km/h [3,4,5,6,7]. In view of the implementation of FG in China, the decision-making process and the derived stop-line crossing behavior of drivers at the change of phases may be inevitably affected, especially at high-speed intersections. Therefore, the driving behavior and safety performance at such intersections calls for reconsideration.

Two important variables characterizing drivers’ stop-line crossing behavior are crossing time and crossing speed [8]. The stop-line crossing time is defined as the elapsed time in seconds after the onset of FG in this study. The stop-line crossing speed is the instantaneous spot speed measured at the stop-line. The former is useful for the estimation of clearance lost time [9] and occurring probability of right-angle traffic conflicts [10,11]; the latter can provide vital information for the estimation of traffic conflict or accident severity [12]. Accurate prediction of the two variables could substantially contribute to the development of on-board safety altering systems or advance warning systems [13,14,15]. It can also facilitate dynamic signal control strategies mitigating traffic conflicts such as the dynamic all-red time extension [16]. Therefore, knowing stop-line crossing time and speed is of great importance for the improvement of the operational and safety performance at signalized intersections.

Many previous studies have addressed the impacts of FG on drivers’ stop-or-go decisions and DZ occurrence. However, few of them focused on the impacts of FG on drivers’ stop-line crossing time and speed, and even less dealt with a phase transition period consisting of a 3 s of FG and a 3 s of Y. The lack of research in this regard is very likely due to the fact that such a design of phase transition period is fairly unique in Chinese cities [1]. 

Hence, the primary objective of this study is to extend the international research community’s understandings on the impacts of the specific combination of FG and Y on stop-line crossing time and speed, based on empirical evidence. The second objective of this study is to compare the stop-line crossing behavior at two different types of intersections implemented with the specific phase transition intervals, i.e., urban intersections and rural high-speed intersections. 

This study collected 1465 high-resolution vehicle trajectories during the phase transition period at five intersections. The selected intersections can be categorized into two groups, i.e., rural high-speed intersections with a speed limit of 80 km/h and urban intersections with a speed limit of 50 km/h. The FG signal setting of both groups is the same and it is the length of Y that distinguishes them. The former group presents a theoretically insufficient length of Y while the latter group shows an appropriate length of Y. The general characteristics of stop-line crossing time and speed of these two groups were first analyzed statistically using the vehicle trajectory data. Then prediction of the stop-line crossing time and speed was performed through a multinomial logit model and a multiple linear regression model respectively.

## 2. Past Research

The following section summarizes pertinent past research regarding the impacts of FG on drivers’ stop-line crossing behavior as well as modelling approaches for drivers’ stop-or-go decisions at the end of the green phase at signalized intersections. 

The use of FG before the Y indication considerably increases the complexity and dynamics of drivers’ decision-making process at signalized intersections [17,18]. A few previous studies have addressed the impacts of FG on drivers’ stop-or-go decisions as well as DZ occurrence and reported distinct results. On the positive side, intersection approaches with a FG signal apparently have a lower proportion of drivers crossing during the red, as compared to those without a FG signal [19,20]. On the other hand, there is a marked increase in the proportion of stopping decisions with the presence of a FG signal [19,21,22]. In addition, the FG signal significantly reduced the area of Type I DZ at the onset of Y, while enlarging the areas of option zone and Type II DZ, as well as inducing conservative stops greatly and aggressive passes slightly [21,23]. The severity of maximum accelerations and decelerations was found to be reduced in the presence of a FG signal. In other words, adding a FG signal is similar to increasing the length of the Y interval [1,20].

Meanwhile, many researchers attempted to model drivers’ stop-or-go decisions at the end of the green phase and they found that it is random and follows a certain probability distribution [24]. Based on the logistic regression models or logit models, such variables as approaching speed, distance or travel time to the stop-line at the onset of Y, vehicle type, driver characteristics, etc. were modeled to contribute to the value of stopping probability [20,21,25,26,27]. In addition, a study used the classification tree model for the correlation between the probabilities of a stop-or-go decision and of red-light-running and the traffic parameters [28]. Meanwhile, Fuzzy Logic theory and Hidden Markov Model theory were also used to interpret drivers’ stop-or-go decision-making process [18,29,30,31]. 

In summary, much attention has been given to the impacts of FG on drivers’ stop-or-go decisions and DZ occurrence. Both positive and negative impacts of FG have been reported in literature and argued. The diversity of conclusions might be due to the length of FG interval, cultural differences, intersection geometries, signal operations and other local conditions. In contrast, little research has addressed the impacts of FG on stop-line crossing speed and time in response to the specific phase transition period including a 3 s of FG and a 3 s of Y. In addition, comparisons of such impacts between the rural high-speed intersections and the urban intersections are in a great shortage. Hence, this study was intended to fill in that research gap. 

## 3. Data Preparation

### 3.1. Site Descriptions

**Figure 1:**
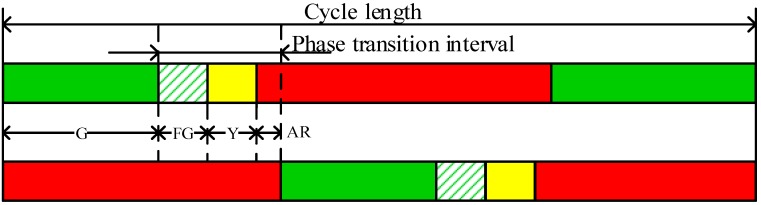
Illustration of the phase transition interval at the study intersections.

Five intersections in Shanghai were selected to collect driver behavior and traffic operation data, which were implemented with a FG of 3 s, a Y of 3 s, and an AR of 1 or 2 s, as shown in Figure 1. The selected intersections can be categorized into two groups, i.e., three rural intersections with a speed limit of 80 km/h and two urban intersections with a speed limit of 50 km/h. Three rural intersections are located on a highway, i.e., Cao’an Road, which is a major corridor connecting the city center and Jiading district. It accommodates a large amount of commuting traffic in the peak hours, including a high percent of large trucks. Two urban intersections are Siping Road and Dalian Road and Rende Road and Jipu Road, where the vast majority of traffic are passenger cars. All the selected intersections are implemented with the red-light-running enforcement cameras. 

It needs to be mentioned that the required Y time for the rural intersections is approximately 5.0 s and that for the urban intersections is 3.0 s, according to the Manual on Uniform Traffic Control Devices (MUTCD) in the United States [3]. Hence, the group of rural intersections can represent those intersections with a FG signal and a theoretically insufficient length of Y, and the group of urban intersections can represent those intersections with a FG signal and an appropriate length of Y. A summary of site conditions at the observed intersections and approaches is provided in Table 1. 

### 3.2. Data Collection and Reduction

The video-taping method was applied to collect the traffic operation and driver behavior data in this study. Video surveys were conducted under sunny weather conditions on normal weekdays in the year of 2013 and 2014. Two high-resolution video cameras were set up on nearby buildings at each intersection approach to record the upstream traffic conditions of the approach and the signal states respectively. An image-processing software with a resolution of 1/30 s (i.e., George 2.1 developed by Nagoya University) was used for data extraction and reduction to ensure high data accuracy. After time synchronization of video data using a stopwatch, calibration was done over the captured global positions through several reference positions, showing that the spatial and temporal trajectory errors were smaller than 0.15 m and 0.1 s respectively. Then vehicles’ positions were recorded every 1/30 s once entering the scope of the video cameras, which was realized manually utilizing George 2.1. Thus we could reproduce the complete vehicular trajectories as well as the corresponding signal states which were also obtained at a time step of 1/30 s through the above preprocessing of raw trajectory data. 

**Figure 2:**
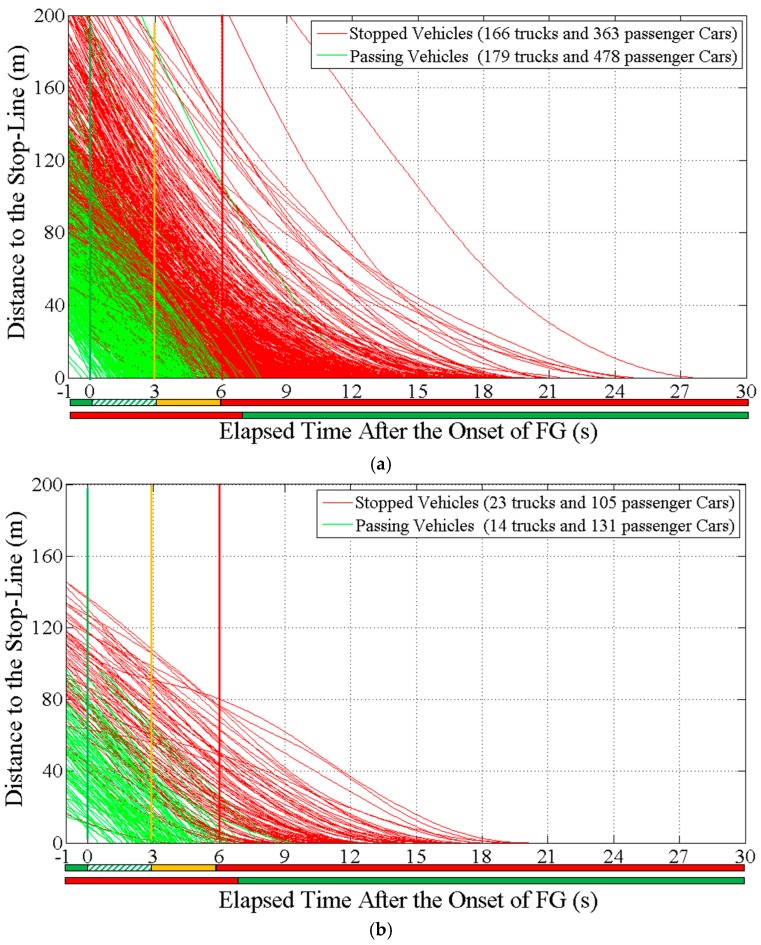
Observed vehicle trajectories at the study intersections; (**a**) Rural intersections (speed limit: 80 km/h); (**b**) Urban intersections (speed limit: 50 km/h).

Only the last-to-stop and the first-to-go through vehicles after the onset of FG that had a following distance greater than 5 s were selected for the analysis to avoid the influence of the presence of leading vehicles. The first-to-stop vehicles refer to the first stopped vehicles in its lane after the onset of FG; the last-to-go vehicles refer to the last passed vehicles in its lane after the onset of FG. Valid sample sizes for the last-to-stop vehicles and the first-to-go vehicles as well as for various stop-line crossing patterns (i.e., crossing during the FG interval (FGC), crossing during the Y interval (YC), red-light-running (RLR)) at each intersection are provided in Table 1. Eventually, 1465 vehicle trajectories including 377 trucks and 1088 passenger cars that encountered the onset of FG were successfully obtained as shown in Figure 2 and used in the following statistical analysis and model development.

## 4. Statistical Characteristics of Stop-Line Crossing Behavior

### 4.1. Statistical Characteristics of Stop-Line Crossing Time

**Figure 3:**
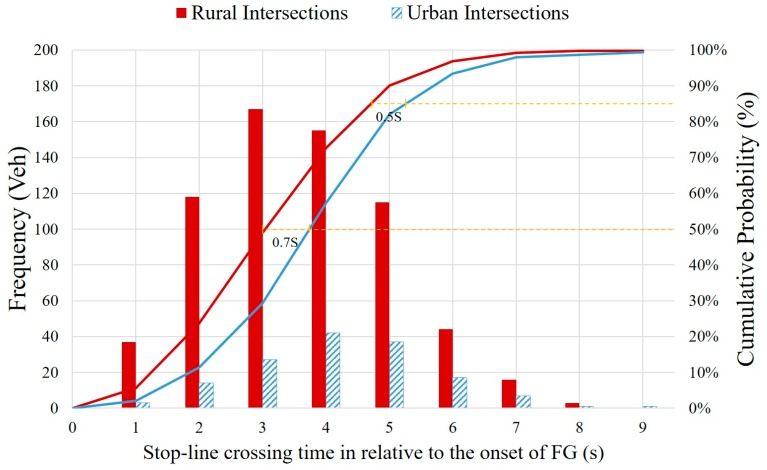
Observed frequencies and cumulative probability of stop-line crossing time.

Stop-line crossing time is defined as the elapsed time in seconds after the onset of FG in this study. It is thus a relative time against the onset time of FG, instead of an absolute time. A stop-line crossing time greater than 6 s (i.e., the sum of the FG and Y time durations) actually translates to a RLR. The observed frequencies and cumulative probability of stop-line crossing time are presented in Figure 3, for the rural intersections and the urban intersections respectively. 

It is shown that the 50th percentile value and the 85th percentile value of the cumulative stop-line crossing time are 3.0 s and 4.7 s at the rural intersections, and those at the urban intersections are 3.7 s and 5.2 s. Though only the first-to-go vehicles were included in the observations of this study, this result can still provide some insights into the unused phase transition time. More specifically, the unused phase transition time could be 3.0 s (i.e., 6.0 s − 3.0 s) at the rural intersections and 2.3 s (i.e., 6.0 s − 3.7 s) at the urban intersections, as far as the first-to-go vehicles are concerned. It reveals that the clearance lost time might be larger at the rural intersections as compared with the urban intersections, given a same signal phase transition setting of a 3.0 s of FG and a 3.0 s of Y. 

In this study, four distinct patterns are defined based on the phase transition intervals to further look into the characteristics of the stop-line crossing time, i.e., Crossing during the FG interval (FGC), Crossing during the Y interval (YC), Red-Light-Running (RLR), and Stop (STOP). Descriptive statistics of the observed approaching speeds and distances to the stop-line at the onset of FG for each of the patterns are presented in Table 2. 

The proportions of the STOP pattern were found to be the largest, which were 44.6% and 46.9% for the rural intersections and the urban intersections respectively, and those of the RLR pattern were found to be the lowest, which were 1.8% and 3.7% respectively. In addition, the proportion of the YC pattern at the urban intersections, i.e., approximately 1/3 out of the total samples, is considerably larger than that at the rural intersections, i.e., around 1/4 out of the total samples. It could be explained by the hypothesis stated earlier in the paper that the use of FG may become more effective in resulting in conservative decisions of drivers at the rural high-speed intersections. More specifically, the use of FG seems to lead to earlier entries (i.e., FGC) or STOP, since the drivers would encounter more severe traffic conflicts if they decided to go but could not pass the intersection during the rest of the time. 

Furthermore, it was found that the mean speed of RLR pattern at rural intersections, i.e., 50.4 km/h, is significantly lower than those of other patterns. This is because all the RLR vehicles (a very low proportion, i.e., 1.8%) were found to be trucks with relatively low running speeds and their drivers are generally more aggressive than the passenger car drivers. 

It can also be seen from the table that the mean of speed at the onset of FG (i.e., *V_FG_*) for the FGC pattern at the rural intersections was the highest, followed by those of the YC pattern, the STOP pattern, and the RLR pattern. Moreover, the mean speed of the RLR pattern was remarkably lower (i.e., about 17%) than those of the other patterns. At a lower speed level, the RLR drivers were supposed to have longer average time to make a stop as compared with those FGC and YC drivers, but they eventually chose to cross. The fact implies that most of the RLRs observed at the rural intersections might be intentional or due to drivers’ decision errors, not because of drivers’ incapability of making a stop. This finding is particularly interesting as it infers that the RLR enforcement cameras are probably less influential at the rural intersections. The differences in the means of *V_FG_* among the four patterns were comparably small at the urban intersections. The mean *V_FG_* of the STOP pattern was approximately 10% lower than those of other patterns, which is rational since the stopping probability of drivers generally increases with the reduced approaching speed. 

In terms of distance to the stop-line at the onset of FG (i.e., *D_FG_*), it was found that the mean of *D_FG_* rose substantially (almost tripled) from the FGC pattern to the STOP pattern for both types of intersections. It reveals that the stopping probability of drivers is strongly associated with the *D_FG_*. Meanwhile, *D_FG_* can be a good explanatory variable for predicting the probabilities of YC and RLR as well.

### 4.2. Statistical Characteristics of Stop-Line Crossing Speed

**Figure 4:**
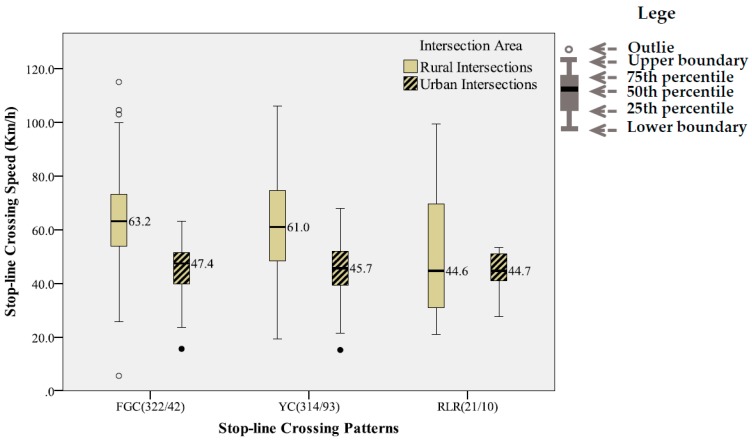
Boxplots of the stop-line crossing speeds for the FGC (crossing during the flashing green interval), YC (crossings during the yellow interval) and RLR (red-light-running) patterns.

Figure 4 further compares the stop-line crossing speed distributions for various stop-line crossing patterns except the pattern of STOP. As shown in the figure, the means of stop-line crossing speed for the patterns of FGC, YC and RLR were very similar for both the rural intersections and the urban intersections. The mean stop-line crossing speeds for the FGC and YC patterns were 63.2 km/h and 61.0 km/h respectively at the rural intersections, about 22% lower than the posted speed limit, i.e., 80 km/h. The corresponding mean speeds were 47.4 km/h and 45.7 km/h at the urban intersections, much closer to the posted speed limit, i.e., 50 km/h.

The mean stop-line crossing speeds for the RLR pattern were almost the same at the two types of intersections, though the posted speed limits differ greatly. Moreover, the discrepancies in the mean stop-line crossing speed between the RLR pattern (i.e., 44.6 km/h) and the other two patterns (i.e., 63.2 km/h and 61.0 km/h) were significant at the rural intersections. However, such a discrepancy was very minor at the urban intersections, i.e., 44.7 km/h versus 47.4 km/h and 45.7 km/h. 

On the other hand, the variance of the stop-line crossing speed was much more considerable at the rural intersections, which could be partly attributed to the traffic compositions, including a high percent of trucks as mentioned earlier.

## 5. Prediction of Stop-Line Crossing Time and Speed 

### 5.1. Prediction of Stop-Line Crossing Time

Stop-line crossing time defined in this study is, on one hand, a continuous and linear increasing variable in nature. On the other hand, it can also be categorized into four stop-line crossing patterns, i.e., YC, FGC, RLR and STOP. Therefore, one modelling approach is considering it as a continuous dependent variable and then using the multiple linear regression models, if it is monotonically increasing for each stop-line crossing pattern. The other approach is converting it to be a multinomial variable and then using the discrete choice models, if it is random and scattered for each stop-line crossing pattern. To select a proper modelling approach, a preliminary analysis on the distribution of stop-line crossing time was firstly conducted to look into whether the stop-line crossing time was significantly differently among the stop-line crossing patterns. The results showed that it was rather random and not monotonically increasing for each stop-line crossing pattern. Therefore, a multinomial logit (MNL) model was finally chosen to predict the stop-line crossing time by considering it as a multinomial variable, i.e., stop-line crossing patterns. 

There are likely to be site-specific factors that influence driver decision-making. To avoid correlations among the factors, the authors used a backward selection process to screen the independent variables. In addition to the driver-specific factors including vehicle type, approaching speed at the onset of FG, and distance to the stop-line at the onset of FG, we firstly included all the site-specific factors including area type, intersection type, observation time periods, and speed limit as well as their joint factors in the model and then eliminated the insignificant variables one by one. Based on the analysis results, the following factors are eventually selected as explanatory variables: vehicle type (*VT*), area type (*AT*), intersection type (*IT*), approach speed at the onset of FG (*V_FG_*), and distance to the stop-line at the onset of FG (*D_FG_*). With respect to the *IT*, the intersections with a size larger than 50 m and with a cycle length greater than 150 s were classified as the large intersections in this study (i.e., Cao’an Road and Jiasongbei Road, Cao’an Road and Xiangjiang Road, and Siping Road andDalian Road listed in Table 1); the rest of the intersections were classified as the small intersections (i.e., Cao’an Road and Caofeng Road and Rende Road and Jipu Road as listed in Table 1). The details of the selected independent variables are explained below.

- *VT* = Vehicle Type, binary variable, 1 = Truck and 0 = Passenger Car;
- *AT* = Area Type, binary variable, 1 = Urban Area and 0 = Rural Area;
- *IT* = Intersection Type, binary variable, 1 = Large Intersection and 0 = Small Intersection;
- *V_FG_* = Speed at the onset of FG (km/h), continuous variable;
- *D_FG_* = Distance to the stop-line at the onset of FG (m), continuous variable.

The MNL model estimation results with the reference category of STOP are presented in Table 3. The MNL model is generally acceptable according to the model statistics, including the Log-likelihood at constant (i.e., 3335.696), the Log-likelihood at convergence (i.e., 1497.164), the McFadden *R*^2^ (i.e., 0.551) and the Hit-ratio (i.e., 87.6%). Based on the estimated model coefficients, the occurring probabilities of the FGC, YC, and RLR patterns against the STOP pattern can then be calculated by Equations (1)–(3) shown below. These equations can then be used to predict the ratios of various stop-line crossing patterns for a particular vehicle at a particular intersection.

(1) $$log\left[\frac{P\left(y=\mathrm{FGC}\right)}{P\left(y=\mathrm{STOP}\right)}\right]=2.454-0.181VT-0.196AT+0.300IT+0.256V_{FG}-0.316D_{FG}$$

(2) $$log\left[\frac{P\left(y=\mathrm{YC}\right)}{P\left(y=\mathrm{STOP}\right)}\right]=1.156-0.088VT+0.528AT+0.097IT+0.080V_{FG}-0.078D_{FG}$$

(3) $$log\left[\frac{P\left(y=\mathrm{RLR}\right)}{P\left(y=\mathrm{STOP}\right)}\right]=-3.226+0.271VT+0.563AT+1.267IT+0.002V_{FG}-0.011D_{FG}$$

where, *P*(*y* = FGC), *P*(*y* = YC), *P*(*y* = RLR), and *P*(*y* = STOP) are the occurring probabilities of the FGC, YC, RLR, and STOP patterns respectively; the other variables are as defined before. 

It was found that vehicle type (*VT*) is not a significant factor for all the four patterns. The approaching speed at the onset of FG (*V_FG_*) and distance to the stop-line at the onset of FG (*D_FG_*) affected the occurring probabilities of the FGC and YC patterns at a significance level of 0.01. The former variable (*V_FG_*) had a positive effect and the latter variable (*D_FG_*) had a negative effect, i.e., the probabilities of the FGC and YC patterns against the STOP pattern increased as the *V_FG_* increased and the *D_FG_* decreased. In addition, the variable of *AT* positively contributed to the ratio of the YC pattern to the STOP pattern at the significance level of 0.05. It implies that drivers are more likely to choose crossing during the Y interval than taking a stop at the urban intersections, which is consistent with the previous findings presented in Table 2. 

Meanwhile, the RLR pattern seems to be irrelative with most of the factors except intersection type (*IT*). One major reason may be that only a small number of RLR samples were collected in this study as shown in Table 1. It was found that the ratio of RLR to STOP occurs more frequently at the large intersections. This is probably due to that, in order to avoid a long waiting time caused by the long cycle lengths adopted at the large intersections, drivers intended to run into the intersections even if they could not reach the stop-line until the start of the red signal.

### 5.2. Prediction of Stop-Line Crossing Speed

Unlike the stop-line crossing time, the stop-line crossing speed is simply a continuous variable. The statistical analysis results presented in Figure 4 have indicated that it is quite randomly distributed for each of the stop-line crossing patterns of YC, FGC and RLR, particularly at the rural intersections. Meanwhile, its means and variances seem to be related to area type and crossing patterns. 

Therefore, a multiple linear regression (MLR) model was further developed to predict the stop-line crossing speed. A multinomial variable of *CP* representing the stop-line crossing patterns was also incorporated into the MLR model, in addition to those determinants used in the MNL model such as *VT*, *AT*, *IT*, *V_FG_*, and *D_FG_*. A preliminary analysis showed that the variable of *IT* was an insignificant factor that influences the stop-line crossing speed. Thus, it was excluded from the model development. The estimated coefficients of the final model are summarized in Table 4, with a total sample size of 802 and a regression *R*^2^ of 0.579. Based on the estimated model coefficients, the stop-line crossing speed can then be formulated by Equation (4) shown below.

(4) $$y=41.749-4.407VT-12.247AT+0.212D_{FG}+0.336V_{FG}-5.925CP$$

where, *y* = stop-line crossing speed (km/h); *CP* = stop-line crossing patterns, which is a multinomial variable, i.e., 1 = FGC, 2 = YC, and 3 = RLR; the other variables are defined as before. 

It can be found that all five independent variables were associated with the stop-line crossing speed at the significance level of 0.01. The impacts of *V_FG_* and *D_FG_* were positive and those of *VT*, *AT*, and *CP* were negative. The results support that the greater the approaching speed and the distance to the stop-line are, the higher the stop-line crossing speed is. Furthermore, stop-line crossing speed tends to be significantly higher for rural intersections, passenger cars, and the FGC pattern. These findings are easy to understand if considering the site conditions presented in Table 1.

## 6. Conclusions

This study empirically analyzed and modeled drivers’ stop-line crossing behavior at signalized intersections in China, where a specific phase transition period consisting of a 3 s of FG and a 3 s Y is commonly applied. Comprehensive statistical analyses were conducted to look into the characteristics of stop-line crossing time and speed, based on 1465 high-resolution vehicle trajectories collected at five intersections in Shanghai. A MNL model and a MLR model were then developed to predict the stop-line crossing patterns (i.e., FGC, YC, RLR, and STOP) and speed, respectively. Major findings of this study are summarized as follows.

- Compared with the rural intersections, the urban intersections had a higher ratio of stop-line crossings during the Y interval and an approximately 0.7 s longer stop-line crossing time which is defined as the elapsed time after the onset of FG.
- Not only approaching speed and distance to the stop-line at the onset of FG, but also area type, imposed a significant influence on the ratios of the FGC and YC patterns to the STOP pattern. Area type also positively contributed to the ratio of the YC pattern to the STOP pattern; in addition, the ratio of RLR to STOP was higher at the large intersections with a long cycle length.
- The larger the approaching speed and the distance to the stop-line were, the higher the stop-line crossing speed was. Stop-line crossing speed was also significantly higher for the rural intersections, the passenger cars, and the FGC pattern.

The findings of this study are useful for the evaluation of the operational and safety performance during the phase transition period at signalized intersections implemented with a FG signal. They could also contribute to the development of safety warning systems as well as dynamic signal control strategies mitigating traffic conflicts. 

However, it must be mentioned that the conclusions based on the five intersections may not hold true for all urban and rural intersections. Distinct driver behavior might be observed at other types of intersections with different traffic demand, size, speed limits, and phase transition intervals. Moreover, the effect of time of day on the drivers’ stop-line crossing behavior was not considered in this study. Thus, it is essential to collect empirical data for other traffic conditions in order to more comprehensively investigate stop-line crossing behavior. In addition, other modelling approaches can also be studied in order to incorporate the prediction processes of stop-line crossing time and speed and to improve the prediction accuracy in the future. For instance, a nested logit model with a top-level model distinguishing stop-or-go and then a lower level model distinguishing among the three “go” alternatives might provide a good alternative. Other random effects models or panel structures can be investigated to eliminate the correlations among the site-specific factors. 

## Figures and Tables

**Table 1:** Summary of site conditions at the observed intersections and approaches.

Intersections	Cao’an Road & Jiasongbei Road	Cao’an Road & Xiangjiang Road	Cao’an Road & Caofeng Road	Siping Road & Dalian Road	Rende Road & Jipu Road
Area Type	Rural Area			Urban Area	
Speed Limit	80 km/h			50 km/h	
Approaches	EB	EB	WB/EB	EB	NB
Lane Configuration	L-T-T-T-R	L-T-T-T-R	L-T-T-T-TR	L-L-T-TR	L-TR
Intersection Size	72 m	72 m	48 m	64 m	40 m
Cycle Length	161 s	160 s	104 s	200 s	86 s
Number of Phases	4	4	3	4	2
Green Time	38 s	45 s	45 s	77 s	45 s
Flashing Green Time	3 s	3 s	3 s	3 s	3 s
Yellow Time	3 s	3 s	3 s	3 s	3 s
All-Red Time	1 s	1 s	1 s	2 s	1 s
Observation Time Periods	12 AM Peak Hours and 6 PM Off-Peak Hours	4 AM Peak Hours and 4 PM Off-Peak Hours	8 AM Peak Hours	8 PM Peak Hours	2 AM Peak Hours and 8 PM Off-Peak Hours
First-to-Go Vehicles (Passenger Cars/Trucks)	201 (156/45)	153 (119/34)	165 (115/50)	177 (127/50)	112 (103/9)
Last-to-Stop Vehicle (Passenger Cars/Trucks)	156 (111/45)	101 (77/24)	272 (175/97)	75 (68/7)	53 (37/16)
FGC (Passenger Cars/Trucks)	111 (85/26)	62 (49/13)	58 (35/23)	91 (63/28)	40 (38/2)
YC (Passenger Cars/Trucks)	83 (65/18)	82 (64/18)	104 (78/26)	84 (63/21)	63 (58/5)
RLR (Passenger Cars/Trucks)	7 (6/1)	9 (6/3)	3 (2/1)	2 (1/1)	9 (7/2)

Note that, in the table, EB = east-bound; WB = west-bound; NB = north-bound; L = exclusive left-turn lane; T = through lane; R = exclusive right-turn lane; TR = shared through and right-turn lane; Intersection size = the distance between the opposite approach stop-lines; FGC = Crossings during the FG interval; YC = Crossings during the Y (yellow) interval; and RLR = Red-Light-Running; FG = Flashing green.

**Table 2:** Descriptive statistics of distances to the stop-line and speeds at the onset of FG for various stop-line crossing patterns.

Intersection Area Types	Variables	Statistical Parameters	Stop-Line Crossing Patterns			
			FGC	YC	RLR	STOP
Rural Intersections (Speed Limit: 80 km/h)	*D_FG_*, m	Mean	35.0	72.0	92.1	104.8
		Standard Deviation	15.3	20.4	22.6	37.2
		Min	3.2	6.3	49.8	23.1
		Max	98.1	125.6	132.9	217.7
		# (%)	322 (27.2%)	314 (26.5%)	21 (1.8%)	529 (44.6%)
	*V_FG_*, km/h	Mean	63.4	61.8	50.4	59.3
		Standard Deviation	15.1	17.6	23.9	18.9
		Min	5.6	19.4	20.9	16.7
		Max	115.0	106.2	99.3	118.9
		# (%)	322 (27.2%)	314 (26.5%)	21 (1.8%)	529 (44.6%)
Urban Intersections (Speed Limit: 50 km/h)	*D_FG_*, m	Mean	28.8	52.6	80.2	95.8
		Standard Deviation	14.0	15.0	16.2	26.9
		Min	7.5	20.9	53.2	39.8
		Max	82.8	93.6	97.6	163.2
		# (%)	42 (15.4%)	93 (34.1%)	10 (3.7%)	128 (46.9%)
	*V_FG_*, km/h	Mean	45.9	45.5	44.4	39.2
		Standard Deviation	10.3	10.4	7.6	8.7
		Min	15.6	15.5	27.7	16.4
		Max	63.1	68.0	53.3	64.5
		# (%)	42 (15.4%)	93 (34.1%)	10 (3.7%)	128 (46.9%)

Note that: FGC = Crossing during the flashing green interval; YC = Crossing during the yellow interval; RLR = Red-light-running; and STOP = stop.

**Table 3:** MNL model estimation results for the prediction of stop-line crossing patterns.

Variables	FGC		YC		RLR	
	B	Sig.	B	Sig.	B	Sig.
Constant	2.454 ***	0.001	1.156 ***	0.003	−3.226 ***	0.001
Vehicle Type, VT	−0.181	0.638	−0.088	0.656	0.271	0.524
Area Type, AT	−0.196	0.654	0.528 **	0.020	0.563	0.200
Intersection Type, IT	0.300	0.364	0.097	0.568	1.267 *	0.008
Speed at the Onset of FG (km/h), *V_FG_*	0.256 ***	<0.001	0.080 ***	<0.001	0.002	0.865
Distance to the Stop-Line at the Onset of FG (m), *D_FG_*	−0.316 ***	<0.001	−0.078 ***	<0.001	−0.011	0.135
Summary Statistics	Number of observations: 1459 veh; Log-likelihood at constant: 3335.696; Log-likelihood at convergence: 1497.164; McFadden *R*^2^: 0.551; Hit Ratio: 87.6%.					

Note that: B = coefficient; Sig. = significance level; *, **, *** represents the 0.1, 0.05, and 0.01 significance levels respectively.

**Table 4:** Multiple linear regression (MLR) model estimation results for the prediction of stop-line crossing speed.

Variables	B	Standard Error	*t*	Sig.
Constant	41.749 ***	2.102	19.863	0.000
Vehicle Type, VT	−4.407 ***	0.845	−5.215	0.000
Area Type, AT	−12.247 ***	1.02	−12.009	0.000
Distance to the Stop-Line at the Onset of FG (m), *D_FG_*	0.212 ***	0.024	8.798	0.000
Speed at the Onset of FG (km/h), *V_FG_*	0.336 ***	0.028	12.152	0.000
Stop-Line Crossing Patterns, CP	−5.925 ***	1.044	−5.673	0.000

Note that: *** represents a significance level of 0.01.
